# The association between head injury and facial fracture treatment: an observational study of hospitalized bicyclists from a level 1 trauma centre

**DOI:** 10.1007/s00701-024-06019-9

**Published:** 2024-03-12

**Authors:** Pål Galteland, Mats Døving, Ingar Næss, Amer Sehic, Tor Paaske Utheim, Torsten Eken, Nils Oddvar Skaga, Eirik Helseth, Jon Ramm-Pettersen

**Affiliations:** 1 Department of Maxillofacial Surgery, Oslo University Hospital Ullevål, Nydalen, PO Box 4956, NO-0424 Oslo, Norway; 2 Faculty of Medicine, Institute of Clinical Medicine, University of Oslo, Oslo, Norway; 3 Department of Neurosurgery, Oslo University Hospital Ullevål, Oslo, Norway; 4 Faculty of Dentistry, Institute of Oral Biology, University of Oslo, Oslo, Norway; 5 Department of Anaesthesia and Intensive Care Medicine, Oslo University Hospital Ullevål, Oslo, Norway; 6 Department of Research and Development, Division of Emergencies and Critical Care, Oslo University Hospital, Oslo, Norway

**Keywords:** Bicycling, Face, Head injury, Fractures

## Abstract

**Purpose:**

To compare the types of facial fractures and their treatment in bicyclists admitted to a level 1 trauma centre with major and minor-moderate head injury.

**Methods:**

Retrospective analysis of data from bicycle-related injuries in the period 2005–2016 extracted from the Oslo University Hospital trauma registry.

**Results:**

A total of 967 bicyclists with head injuries classified according to the Abbreviated Injury Scale (AIS) were included. The group suffering minor-moderate head injury (AIS Head 1–2) included 518 bicyclists, while 449 bicyclists had major head injury (AIS Head 3–6). The mean patient age was 40.2 years (range 3–91 years) and 701 patients (72%) were men. A total of 521 facial fractures were registered in 262 patients (on average 2 facial fractures per bicyclist). Bicyclists with major head injury exhibited increased odds for facial fractures compared to bicyclists with minor-moderate head injury (sex and age adjusted odds ratio (OR) 2.75, 95% confidence interval (CI) 2.03–3.72, *p* < 0.001. More specifically, there was increased odds for all midface fractures, but no difference for mandible fractures. There was also increased odds for orbital reconstruction in cyclist with major head injury compared to bicyclist with minor-moderate head injury (adjusted OR 3.34, 95% CI 1.30–8.60, *p* = 0.012).

**Conclusion:**

Bicyclists with more severe head injuries had increased odds for midface fractures and surgical correction of orbital fractures. During trauma triage, the head and the face should be considered as one unit.

## Introduction

Bicycling is an efficient mode of transportation, and regular bicycling offers notable health benefits, e.g., a reduced risk of cardiovascular disease, obesity, and cancer [3]. Additional advantages include less air pollution and fewer parking issues [36]. However, bicycling also carries injury risk because riders are vulnerable during accidents. Urban bicycling has the highest risk of injury per kilometer compared to other transportation modes, leading to debates about whether its benefits outweigh the risks [17, 31].

Head and face injuries are common in bicycle accidents [8, 11, 30]. One theory is that bicyclists are often thrown over their handlebars [34]. The facial skeleton can protect the brain by absorbing energy [4, 22, 39]. However, a powerful facial impact can transmit forces to the skull, including the base of the skull, causing brain injury [7, 23, 24, 40]. While traumatic brain injuries are a major cause of death and disability [20], facial fractures are rarely life-threatening, but can diminish the quality of life and place a financial burden on the healthcare system [2].

The association between head injury and facial fractures is complex and potentially bidirectional [11, 18, 28]. The present work aimed to explore variations in facial fractures and their corresponding surgical treatments among bicyclists with minor to moderate head injuries and those with major head injuries. We hypothesized that bicyclists with major head injuries are more likely to require surgical correction of facial fractures than those with minor-moderate head injuries. Unlike most studies that focus on facial fractures as markers of head injury, our research aimed to consider head injury as the predictive factor.

## Methods and materials

Oslo University Hospital Ullevål (OUH-U) serves as a primary trauma hospital for Oslo, a city with more than 700,000 citizens. Additionally, it is the level 1 trauma centre for the South-Eastern region of Norway, which is home to approximately 3.1 million inhabitants. Patients who sustain a potentially serious injury with an estimated transport time of less than 45 min are normally transported directly to OUH-U, as well as those who are obviously in need of urgent neurosurgical care independent of transport time [27]. Patients who do not qualify for direct transport to OUH-U receive initial treatment at other local acute care hospitals or outpatient clinics, before transfer to OUH-U if necessary [37]. During the study duration, 20 acute care hospitals in South-Eastern Norway referred patients to OUH-U. Hence, the present investigation does not represent a population-based study but rather a subset of potentially severely injured patients from Oslo and South-Eastern Norway.

Data from bicycle-related injuries in the period from January 1, 2005, to December 31, 2016, was extracted from the OUH Trauma Registry (TR-OUH). Passengers on a bicycle at the time of the crash and patients declared dead on arrival at OUH-U according to the Utstein template definition were included [35]. Pedestrians struck by a bicycle were excluded, and missing data was not imputed.

The variables included were age, sex, date of injury, Abbreviated Injury Scale (AIS) codes for injuries in the AIS regions Head and Neck and for skeletal injury in the region Face, Injury Severity Score (ISS), 30-day mortality, and surgical procedure codes for treatment of facial fractures. Anatomical injury and severity were classified according to AIS 90 update 98 [12]. All codes describing facial skeletal injuries in the AIS Face region (AIS 25*) were included, except temporomandibular joint injury (AIS 2516*) and tooth injury (AIS 2514*). Injuries were further categorized as fractures of the maxilla (2508*), zygoma (2518*), nose (2510*), orbit (2512*), and mandible (2506*). Orbital roof and frontal bone injuries are part of the AIS region Head.

Surgical treatment was based on NOMESCO Classification of Surgical Procedures (NCSP) codes [29]. The following NCSP codes were included: Orbita (CAC00, CAC 10), nose (DHD10, DHD20, DHD30), zygoma (EEC30, EEC35), maxilla (EEC20, EEC25), and mandible (EDC32, EDC36, EDC38, EDC42). One or more procedures in the same location were counted as one procedure.

Head injury severity was the predictor variable. Injuries with AIS severity codes 1 (minor) and 2 (moderate) were defined as “minor-moderate head injury” and those with severity codes 3–6 (serious, severe, critical, and maximal) as “major head injury.” We analyzed facial fractures and their treatment as the outcome variables, taking into account age and sex for adjustments.

Stata SE 17 (College Station, TX, USA) was used for statistical analysis. Descriptive statistics are presented with absolute number and percentage. Pearson chi-squared test was used to detect differences in categorical variables. For comparing differences in continuous variables, the *T*-test was employed, and in instances where the data distribution was skewed, the Mann–Whitney *U* test was utilized. Multivariable logistic regression analysis was used to control for confounding effects of age and sex. The results from the regression analysis are presented as odds ratio (OR) with 95% confidence interval (CI). Statistical significance was assumed for two-tailed *p* < 0.05.

## Results

**Fig. 1:**
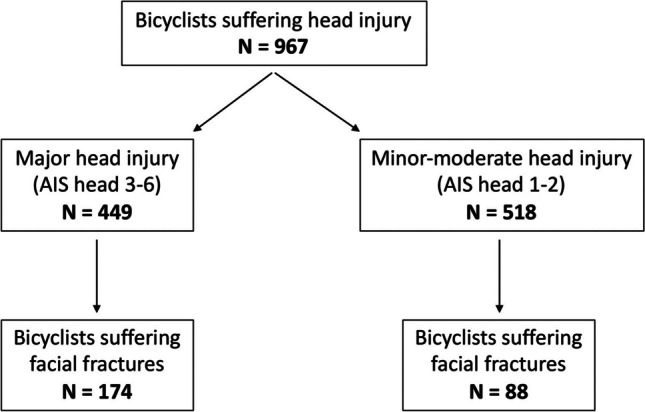
Flowchart of 967 injured bicyclists with head injury admitted to OUH-U from 2005 to 2016

The study included 967 bicyclists with head injury, 72% of whom were men and the mean age was 40.2 years (range 3–91 years). Minor-moderate head injuries were sustained by 54% of the bicyclists, while 46% had major head injuries (Fig. 1). Patients with major head injuries had a higher proportion of facial fractures compared to the minor-moderate head injury group (174/262 versus 88/262, *p* < 0.001). Further analyses revealed a threefold increased odds for facial fractures in the more severe head injury group (OR 3.09, CI 2.30–4.16, *p* < 0.001; sex and age adjusted OR 2.75, CI 2.03–3.72, *p* < 0.001).

Patient characteristics are detailed in Table 1. There was a significant age difference observed between the groups, with individuals having more severe head injuries (AIS Head 3–6) being generally older than those with less severe injuries (AIS Head 1–2). The distribution of sex between the groups showed no significant difference, indicating a similar proportion of males in both the more and less severely injured groups. A notable increase in the Injury Severity Score (ISS) was observed in the group with more severe head injuries, a difference that was statistically significant. Additionally, the mortality rate was substantially higher in the group with more severe injuries.

**Table 1:** Patient characteristics of 967 bicyclists with AIS Head 3–6 and AIS Head 1–2. Age is given in the number of years and 95% confidence interval (CI) is in parenthesis. Mortality is all-case death within 30 days. *T*-test is used for age and ISS. Chi quadrat is used for sex and mortality was analyzed with Fisher’s exact test due to low numbers

Variable	AIS Head 3–6, *N* = 449	AIS Head 1–2, *N* = 518	*p*-value
Age, mean (CI)	43.8 (41.9–45.6)	37.2 (35.6–38.8)	< 0.001
Male, n (%)	335 (74.6)	366 (70.6)	0.170
ISS mean (CI)	22.3 (21.2–23.4)	8.3 (7.7–8.9)	< 0.001
Mortality, *n* (%)	30 (6.7)	1 (0.2)	< 0.001

Table 2 shows the association with different facial fractures for major head injury versus minor-moderate head injury. Of all the facial fractures, 70% (365/521) were observed in bicyclists with major head injuries, who also had increased odds for all midface fractures (orbit, nose, zygoma, and maxilla) compared to the group with minor-moderate head injuries. Midface fractures represented 91% (475/521) of all the facial fractures in patients with major head injuries. There was no difference in the odds for mandible fracture between the two groups.

**Table 2:** Odds ratios (ORs) with 95% confidence interval (CI) for the different facial fracture types for bicyclists with major head injury versus minor-moderate head injury. The ORs are presented both unadjusted and adjusted for age and sex. A total of 521 facial fractures were seen in 262 patients

	Number of fractures, *N* = 521	AIS Head 3–6, *N* = 365	AIS Head 1–2, *N* = 156	OR (CI)	*p*-value	Adjusted OR (CI)	Adjusted *p*-value
Orbit	127	96	31	4.27 (2.79–5.55)	< 0.001	3.72 (2.41–5.74)	< 0.001
Nose	77	48	29	2.02 (1.25–3.26)	< 0.010	1.81 (1.11–2–96)	0.017
Zygoma	117	87	30	3.90 (2.53–6.05)	< 0.001	3.42 (2.19–5.33)	< 0.001
Maxilla	154	107	47	3.14 (2.17–4.54)	< 0.001	2.67 (1.83–3.90)	< 0.001
Mandible	46	27	19	1.68 (0.92–3.06)	0.091	1.64 (0.89–3.02)	0.113

A total of 85 patients underwent surgery for 134 facial fractures. Among these patients, 33 (39%) had minor-moderate head injury, while 52 (61%) had major head injury. Bicyclists with major head injury had a greater likelihood of undergoing surgical treatment for facial fractures than those with mild to moderate injuries (OR 2.91, CI 2.22–3.80, *p* < 0.001; sex and age adjusted OR 2.79, CI 2.13–3.66, *p* < 0.001).

**Fig. 2:**
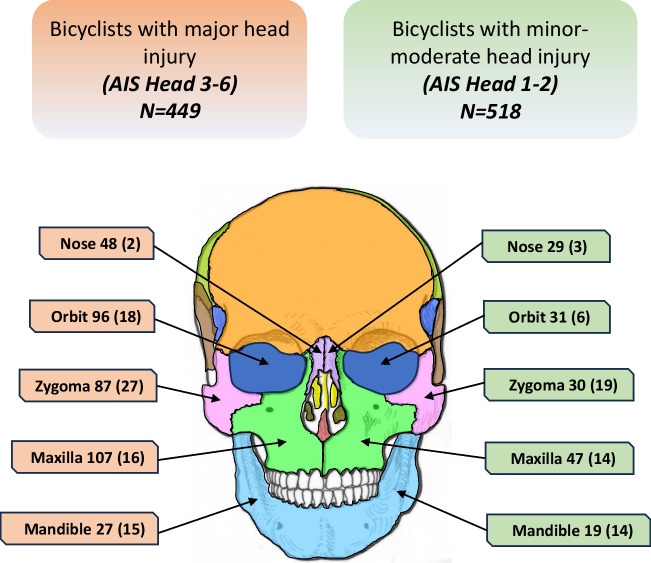
The correlation between the location of facial fractures, including those surgically treated, and the severity of head injury. It compares patients with major head injuries (AIS Head 3–6) against those with minor to moderate head injuries (AIS Head 1–2). Displayed in the figure is the tally of different facial fractures, with the number requiring surgical treatment specified in parentheses

Table 3 shows the ORs for surgical interventions for different types of facial fractures among hospitalized bicyclists, comparing the major head injury group to the minor-moderate injury group. Surgical intervention for midface fractures accounted for 78% (105/134) of all facial fracture treatments. Of the fractures treated, 75% (42/56) were in the midface for those with minor-moderate head injury, compared to 81% (63/78) for those with major head injury (Fig. 2). There was a more than threefold increase in the odds for surgical treatment of orbital fractures in bicyclists with major head injury. However, there were no statistically significant differences between the two groups for the treatment of all other fracture sites.

**Table 3:** Odds ratios (ORs) with 95% confidence interval (CI) for surgical treatment of facial fractures in bicyclists with major head injury versus minor-moderate head injury, unadjusted and adjusted for sex and age. Due to the small sample size, Fisher’s exact test was used to analyze the surgical treatment of nose fractures, but CI and adjusted *p*-value were not available (N/A) due to insufficient data

	Number of fractures, *N* = 134	AIS Head 3–6, *N* = 78	AIS Head 1–2, *N* = 56	OR (CI)	*p*-value	Adjusted OR (CI)	Adjusted*p*-value
Orbit	24	18	6	3.56 (1.40–9.06)	< 0.01	3.34 (1.30–8.60)	0.012
Nose	5	2	3	N/A	0.57	N/A	N/A
Zygoma	46	27	19	1.68 (0.92–3.06)	0.09	1.50 (0.81–2.78)	0.200
Maxilla	30	16	14	1.33 (0.64–2.76)	0.44	1.14 (0.54–2.40)	0.730
Mandible	29	15	14	1.24 (0.59–2.61)	0.56	1.23 (0.58–2.61)	0.590

## Discussion

In this retrospective study from a level 1 trauma centre, we observed that more severe head injuries in bicyclists were associated with a higher proportion of midface fractures compared to mild-moderate head injuries. However, there was no significant difference in the odds of mandible fracture between the two groups. Furthermore, more severe head injury was associated with an increased likelihood of receiving surgical treatment for facial fractures. However, upon closer examination, this was found to be statistically significant only for surgical treatment of orbital fractures.

Head injury and facial fractures often coincide, as the forces that cause facial fractures can also have the potential to damage the brain through various mechanisms, such as energy transmission, acceleration-deceleration, and rotational forces [16, 25]. A linear skull fracture, particularly involving the base of the skull, can transmit forces that cause facial fractures.

In general, high-energy trauma is more likely to cause a combination of head and facial injury, compared to a trauma with low energy [13, 24]. Fractures that involve the frontal bone or midface have a higher risk of head injury due to the proximity of these structures to the brain [1, 33]. This is in line with our results, as we found increased odds for midfacial fractures among patients with major head injuries. In addition, surgical treatment of orbital fractures was strongly associated with the occurrence of major head injury. This may indicate a higher impact energy and transmission of energy from the orbit to the neurocranium and brain.

Keenan et al. studied bicyclists and the association between head injury and facial fractures in a patient population similar to ours [19]. They found a strong association between facial fractures and brain injury, indicating that facial fractures serve as markers of head injury. The number of facial fractures was lower compared to our study, but they similarly observed a higher incidence of orbit fractures compared to mandible fractures.

Our study is in partial alignment with a recent systematic review and meta-analysis which also found an association between head injuries and facial fractures, but reported mandible fractures as the most frequent [32]. This study from Othman et al. incorporated data from all trauma mechanisms, not only bicyclists. The variation in trauma mechanisms can affect the types and frequencies of the observed injuries. It was also evident that the study reported mandible fractures to be predominant in Africa and Asia indicating a geographical variance, possibly influenced by the prevalence of motor vehicle accidents, transportation modes, and cultural factors [21].

When an individual experiences a head injury, it is of utmost importance for the attending healthcare provider to thoroughly assess for potential indicators of facial fractures. Indicators can include pain in the facial region, swelling, or any discernible deformities [38]. In a similar way, if an individual presents with a facial fracture, it is crucial to evaluate for accompanying signs and symptoms that might point toward a head injury. This would encompass symptoms like a loss of consciousness, disorientation or confusion, and any noticeable neurological deficits or changes in behaviour [6]. Such a thorough assessment is vital to ensure comprehensive patient care and to address all potential complications stemming from the injury.

Prompt identification and treatment of both head injury and facial fractures are important to prevent further damage and to ensure the best possible outcome. Our study indicates that for patients with more severe head injuries, a low threshold should be maintained for conducting facial CT scan, and this is in line with other studies [14, 15, 26]. The treatment of facial fractures often involves open reduction and rigid fixation [5]. Management of head injury may involve acute cranial neurosurgery, neurointensive care, close monitoring for signs of neurological worsening, and neurorehabilitation [10]. Treatment of craniomaxillofacial injuries requires collaboration between several specialties, including neurosurgeons and maxillofacial surgeons for optimal outcomes.

Our results demonstrate that neurosurgeons must pay attention to the potential presence of concomitant facial fractures in patients with major head injuries, with a particular emphasis on the heightened risk of orbital fractures necessitating reconstructive surgery. While the assessment and management of traumatic head injuries should be prioritized, our data suggest that, in cases of orbital fractures requiring open surgical intervention, scheduling the procedure within 2 weeks of the injury is generally feasible without compromising patient outcomes, unless patients are presenting retrobulbar hematoma or muscle entrapment in the paediatric patient, conditions that necessitate urgent management [9]. This approach allows for the prioritization of immediate life-threatening conditions while ensuring timely treatment of significant orbital injuries.

A limitation of the present study is the focus on bicyclists in our trauma registry. As a result, many bicyclists with facial fractures who were admitted to the hospital more than 24 h after the injury, had sustained minor injuries, and/or were not received by a trauma team were not included in our analysis. Therefore, the identified relationship between facial fractures and head injuries should not be applied indiscriminately to the broader population of bicyclists who may experience facial fractures from other causes. Furthermore, the findings cannot be generalized to other injury mechanisms, such as motor-vehicle accidents and assaults.

## Conclusion

This observational study shows that bicyclists with more severe head injury had higher odds for midface fractures and a higher likelihood of orbital reconstruction compared to bicyclists with less severe head injury. The findings underscore the close association between head and facial injury, and that the head and face should be considered as one unit in high-energy trauma.

## Data Availability

All data and material are available upon request.

## Code availability

This declaration is not applicable.
